# Seroprevalence and Risk Factors of Hepatitis B Virus Among Newly Diagnosed Cancer Patients in Khartoum State: Implications for Chemotherapy Management and Screening Protocols

**DOI:** 10.1002/jgh3.70171

**Published:** 2025-04-29

**Authors:** Anwaar Abdulgader Merghani Alkhdir, Abubaker A. Mohamedsharif, Isra Bdraldein Salih Mohammed, Amin Mohamed Abbas

**Affiliations:** ^1^ Internal Medicine Department Faculty of Medicine, University of Bahri Alkadroo Sudan; ^2^ Faculty of Medicine, University of Khartoum Khartoum Sudan; ^3^ Ibn Sina Specialized Hospital Khartoum Sudan

**Keywords:** blood transfusion, cancer, chemotherapy, HBV screening, hepatitis B virus, oncology, seroprevalence, Sudan

## Abstract

**Aims:**

This study aimed to determine the seroprevalence of hepatitis B virus (HBV) among newly diagnosed cancer patients in Khartoum State, Sudan, prior to chemotherapy initiation and to identify associated risk factors.

**Methods and Results:**

A cross‐sectional study was conducted from October 2022 to April 2023 at various oncology centers in Khartoum State. A total of 300 newly diagnosed cancer patients, aged 18 years and older, were included. Blood samples were screened for Hepatitis B surface antigen (HBsAg) using a rapid immunochromatographic test (ICT) and confirmed by enzyme‐linked immunosorbent assay (ELISA). The study found that 31 patients (10.3%) were HBsAg positive. A significant association was observed between HBV positivity and patients' history of blood transfusions (41.9% of positive cases), as well as geographic origin, with higher rates among those from Western Sudan (44.7%) and Central Sudan (40.6%). Patients diagnosed with hematological malignancies exhibited the highest HBV prevalence. Statistical analysis revealed significant correlations between HBV positivity and factors such as age, gender, residence, and transfusion history, indicating these as key risk factors.

**Conclusion:**

The study reveals a notable HBV seroprevalence among cancer patients in Khartoum, particularly linked to blood transfusion history and specific regions. These findings emphasize the need for routine HBV screening in oncology patients before chemotherapy to prevent reactivation and improve clinical outcomes.

## 1 Introduction

Hepatitis B virus (HBV) infection is a major global health issue, with approximately 290 million individuals infected worldwide. The World Health Organization (WHO) has set ambitious targets for the elimination of HBV by 2030, recognizing the urgent need to address this public health threat [1]. According to the Global Burden of Disease study, viral hepatitis is responsible for around 1.34 million deaths annually, which is comparable to the mortality rates associated with HIV/AIDS (1.3 million), malaria (0.9 million), and tuberculosis (1.3 million) [2]. The mortality due to viral hepatitis has increased by 63% since 1990, and it is currently ranked as the seventh leading cause of death globally [3]. Despite this alarming trend, there remains a significant gap in awareness and action to combat HBV infection.

In Sudan, the prevalence of HBV is notably high, with studies indicating that it is the leading cause of chronic liver disease and hepatocellular carcinoma (HCC) [4]. Approximately 6.1% of the adult population in Sub‐Saharan Africa is infected with chronic HBV, with Sudan contributing significantly to this burden [5]. The high prevalence can be attributed to factors such as socio‐economic conditions, lack of awareness, and inadequate vaccination coverage. The healthcare system in Sudan faces considerable challenges in managing HBV infections, particularly among vulnerable populations such as cancer patients undergoing chemotherapy.

HBV can manifest as either an acute or chronic infection. Chronic HBV infection progresses through three distinct phases: the immune tolerance phase, the immune clearance phase with HBeAg seroconversion, and the inactive phase [6]. During the inactive phase, there is a risk of reactivation, especially in immunocompromised individuals, such as those receiving chemotherapy. Reactivation of HBV can lead to severe complications, including liver failure, which poses significant risks for patients undergoing cancer treatment [7].

The relationship between HBV and cancer is well‐documented. Chronic HBV infection is associated with an increased risk of developing HCC and other malignancies, primarily due to the immune system's failure to control the virus [8]. Cancer patients, particularly those receiving immunosuppressive therapies, are at a heightened risk for HBV reactivation. This necessitates the importance of screening for HBV prior to initiating chemotherapy, as early detection can facilitate timely antiviral prophylaxis and reduce the risk of severe complications [9].

Screening for HBV among cancer patients is critical. Leading health organizations, including the American Association for the Study of Liver Diseases (AASLD), the European Association for the Study of the Liver (EASL), and the Asian Pacific Association for the Study of the Liver (APASL), recommend universal screening for HBV in patients who are about to receive chemotherapy [10]. However, the American Society of Clinical Oncology (ASCO) suggests that screening should be limited to high‐risk populations, leading to a lack of consensus in clinical practice guidelines. This divergence in recommendations may stem from concerns regarding the cost‐effectiveness of universal screening, which can create confusion among healthcare providers [11].

Several risk factors have been identified that contribute to HBV reactivation during chemotherapy. High viral load, HBeAg positivity, use of steroids, younger age, male gender, and specific types of cancer, such as lymphoma and breast cancer, are significant predictors of reactivation [12]. Understanding these risk factors is essential for developing targeted screening strategies and prophylactic measures for at‐risk populations.

In conclusion, the high prevalence of HBV in Sudan, particularly among newly diagnosed cancer patients, underscores the urgent need for effective screening and management strategies. The potential for HBV reactivation during chemotherapy poses a serious threat to patient safety and treatment outcomes. By implementing universal screening protocols and providing appropriate antiviral prophylaxis, healthcare providers can mitigate the risks associated with HBV reactivation, ultimately improving the prognosis for cancer patients in Khartoum State and beyond.

## 2 Materials and Methods

### 2.1 Study Design

This research was designed as a descriptive cross‐sectional study, aiming to capture a snapshot of the prevalence of HBV infection among newly diagnosed cancer patients. By focusing on a single point in time, the study explored potential correlations with factors such as age, gender, and geographical distribution, providing valuable insights into the health status of this specific patient group.

### 2.2 Study Area

The study was conducted across multiple oncology centers in Khartoum State, Sudan. These centers were selected due to their high patient volumes and the comprehensive cancer treatment services they provide, including chemotherapy. This setting was ideal for capturing data from a diverse group of cancer patients.

### 2.3 Study Duration

Data collection took place over a 7‐month period, from October 2022 to April 2023. This timeframe allowed for the collection of extensive data from newly diagnosed cancer patients, ensuring a robust analysis of the prevalence of HBV infection and its association with various demographic and clinical factors.

### 2.4 Study Population

The study targeted adult cancer patients, aged 18 years and older, who were newly diagnosed and attending oncology centers in Khartoum State for chemotherapy. This population was chosen to understand the prevalence of HBV among patients in the early stages of cancer treatment.

### 2.5 Sample Size and Sampling Technique

The study aimed to include all newly diagnosed cancer patients who attended oncology centers in Khartoum State during the study period. An estimated sample size of 300 patients was targeted, based on the anticipated number of new patients seen weekly across multiple outpatient clinics. This approach ensured a comprehensive representation of the patient population within the study timeframe.

### 2.6 Inclusion Criteria

To be included in the study, participants had to be 18 years of age or older, of any gender, newly diagnosed with cancer, and scheduled for chemotherapy. These criteria ensured that the study focused on adults at the beginning stages of cancer treatment.

### 2.7 Exclusion Criteria

Participants were excluded if they were under 18 years of age, planned for treatment modalities other than chemotherapy, had mental disabilities, or had a known positive hepatitis B status. These exclusions helped maintain a focus on the intended patient population for the study.

### 2.8 Data Collection and Laboratory Processing

Data were collected through a combination of a structured, interview‐based questionnaire, and laboratory testing. The questionnaire, which was pre‐tested and coded, gathered demographic and clinical information from participants. Blood samples were collected under aseptic conditions, with 5 mL of venous blood drawn and placed in anticoagulant‐containing collection containers. The serum was tested for hepatitis B virus surface antigen (HBsAg). Initially, a rapid immunochromatographic test (ICT) was used. Non‐reactive results indicated HBsAg‐negative status, while reactive results were further tested using enzyme‐linked immunosorbent assay (ELISA). Serum samples reactive in both tests were classified as HBsAg positive.

### 2.9 Study Variables

The dependent variables included the type of malignancy and HBV status. The independent variables were age, gender, and geographical distribution. These variables were selected to explore their potential relationships and effects within the study population.

### 2.10 Data Analysis

Data from all participants were entered into the Statistical Package for Social Sciences (SPSS) for analysis. The analysis focused on summarizing demographic and clinical data and examining relationships between HBV status and other variables such as age, gender, and type of cancer. Descriptive statistics were used to present the findings, and further statistical analyses were performed to identify any significant correlations.

## 3 Results

### 3.1 Demographic and Clinical Characteristics of Participants

A total of 300 newly diagnosed cancer patients planned for chemotherapy participated in this cross‐sectional study conducted from October 2022 to April 2023 in Khartoum State, Sudan. The study population consisted of 143 men (47.7%) and 157 women (52.3%). The mean age of participants was predominantly between 41 and 60 years, accounting for 146 individuals (48.7%), while the age distribution included 63 patients (21%) aged 18–40 and 91 patients (30.3%) aged 61–90 (see Table 1).

**TABLE 1:** Age distribution among study participants, *n* = 300.

Age group in years	Frequency	Percentage
18–40	63	21
41–60	146	48.7
61–90	91	30.3

**FIGURE 1:**
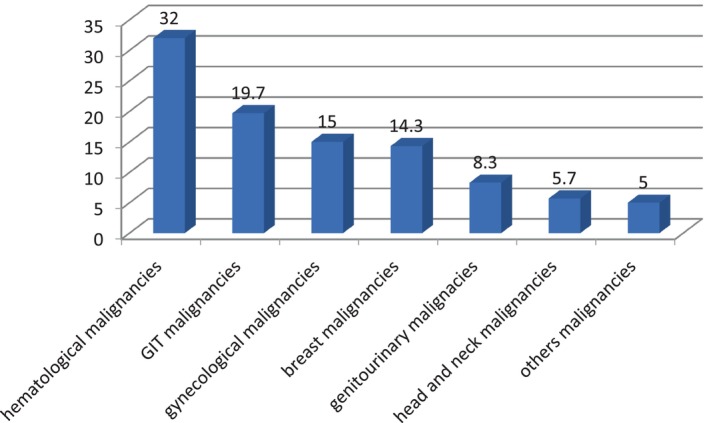
Malignancies distribution among study participants, *n* = 300.

Geographically, 134 participants (44.7%) were from Western Sudan, followed by 122 (40.6%) from Central Sudan. The most common malignancies diagnosed were hematological malignancies in 96 patients (32%), followed by gastrointestinal (GIT) malignancies in 59 patients (19.7%), gynecological malignancies in 45 patients (15%), breast malignancies in 43 patients (14.3%), and genitourinary malignancies in 25 patients (8.3%). Additionally, 32 patients (10.7%) had other types of malignancies (see Figure 1).

### 3.2 Seroprevalence of HBV

**FIGURE 2:**
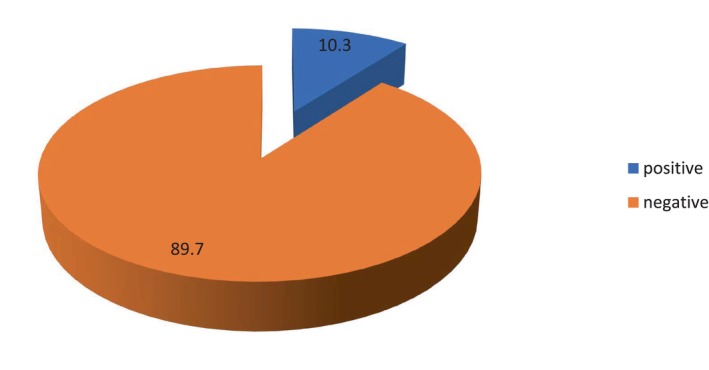
HBsAg (ICT) status distribution among study participants, *n* = 300.

Of the 300 participants, 31 (10.3%) tested positive for HBsAg using the ICT. All participants who tested positive by ICT were also confirmed positive by the ELISA test (see Figure 2).

### 3.3 Risk Factors Associated With HBV Positivity

Among the 31 HBsAg‐positive participants, the most common risk factor was a history of blood transfusion, with 13 patients (41.9%) having received transfusions. Ten patients (32.2%) reported no known risk factors. Other risk factors included a household member infected with HBV in five patients (16.1%) and a history of hemodialysis in two patients (6.6%). No participants reported a history of drug injection or organ donation (see Table 2).

**TABLE 2:** Risk factors distribution among study participants, *n* = 31.

Risk factors	Frequency	Percentage	*p*
Household member with HBV	5	16.1	< 0.001
Hemodialysis	2	6.4	0.08
Blood transfusion	13	41.6	< 0.001
None	10	32.3	—

### 3.4 Statistical Analysis

The statistical analysis aimed to evaluate the associations between HBV seropositivity and various demographic and clinical factors among the 300 participants in this study. Chi‐square tests were employed to assess the significance of associations between categorical variables.

#### 3.4.1 Age and Malignancy Associations

Among patients diagnosed with blood malignancies, a significant association was observed between age and HBV seropositivity. Specifically, among patients aged 41–60 years, 5 out of 146 (3.4%) were HBsAg positive, compared to 12 out of 63 (19.0%) in the 18–40 age group, indicating that younger age (18–40 years) is significantly associated with increased HBV seropositivity (*p* = 0.01). In contrast, among patients with breast malignancies, the association between age and HBV seropositivity was not statistically significant (*p* = 0.10), suggesting that further investigation is required to clarify this relationship.

#### 3.4.2 Geographic and Cancer‐Type Associations With HBV Seropositivity

The analysis demonstrated a significant association between geographic origin (Western/Central Sudan) and HBV seropositivity (*p* = 0.02). Among the 31 HBsAg‐positive participants, 14 (45.2%) were from Western Sudan and 12 (38.7%) from Central Sudan. Conversely, the association between residence and breast malignancies was significant (*p* < 0.001). Of the 43 patients with breast cancer, 7 (16.3%) were HBsAg positive, compared to 24/257 (9.3%) in other cancer types, indicating regional epidemiological variations in HBV prevalence.

#### 3.4.3 Gender and HBV Positivity

The analysis revealed a significant association between gender and HBV seropositivity, with a *p*‐value of 0.00. Among the 143 male participants, 8 (5.6%) tested positive for HBsAg, while 23 out of 157 female participants (14.6%) were positive. This indicates a higher prevalence of HBV infection among female patients compared to male patients.

#### 3.4.4 Risk Factors and HBV Infection

A significant association was observed between HBsAg positivity and a history of blood transfusion (41.9%, *p* < 0.001). Thirteen out of 31 HBsAg‐positive participants (41.9%) reported having received transfusions, with a chi‐square test yielding a *p*‐value of less than 0.001. This strong correlation highlights the importance of blood transfusion as a significant risk factor for HBV infection.

Additionally, the presence of a household member infected with HBV was significantly associated with HBsAg positivity, with 5 out of 31 positive participants (16.1%) reporting this risk factor (*p* = 0.00). This finding emphasizes the potential for familial transmission dynamics in the epidemiology of HBV among cancer patients.

In summary, the statistical analysis demonstrated significant associations between HBV seropositivity and various demographic factors, including age, gender, and residence, as well as specific risk factors such as blood transfusions and household exposure to HBV. While some associations were not statistically significant, the overall findings underscore the need for targeted screening and preventive measures for high‐risk groups, particularly those with a history of blood transfusions and familial HBV exposure. Further research is warranted to explore the underlying mechanisms and to develop effective interventions for HBV prevention in cancer patients.

## 4 Discussion

The seroprevalence of HBV among newly diagnosed cancer patients in Khartoum State, Sudan, highlights a significant public health concern, particularly in the context of chemotherapy treatment. In this cross‐sectional study conducted from October 2022 to April 2023, a seroprevalence rate of 10.3% was observed among 300 participants, which aligns with findings from other studies in regions with similar HBV prevalence rates [13]. This prevalence underscores the need for routine HBV screening and preventive measures in oncology settings, especially given the potential for HBV reactivation during immunosuppressive therapy [14].

Demographic analysis revealed that the majority of participants were female (52.3%), with a significantly higher seroprevalence of HBV among women (14.6%) compared to men (5.6%). This gender disparity is noteworthy, as it contrasts with some previous research suggesting higher rates of HBV among males due to riskier behaviors [1]. However, recent studies indicate a rising trend of HBV infections among women, possibly linked to increased healthcare access for antenatal services where screening is more prevalent [15]. This shift necessitates a reevaluation of gender‐specific risk factors and healthcare practices regarding HBV transmission.

Age distribution among the participants showed that the highest prevalence of HBV was in the 41–60 age group (48.7%), which is consistent with global trends indicating that HBV infection rates peak among middle‐aged adults [16]. The findings suggest that chronic HBV infections often remain asymptomatic until complications arise, particularly in older adults [17]. Furthermore, geographical analysis indicated higher HBV prevalence in Western Sudan (44.7%) and Central Sudan (40.6%), reflecting regional disparities in healthcare access and HBV transmission dynamics [6]. Such geographic variations emphasize the importance of localized public health strategies to address HBV infection.

Despite mandatory HBsAg screening for blood donors in Sudan, the strong association between transfusion history and HBV positivity (41.9%, *p* < 0.001) suggests gaps in protocol adherence or test sensitivity. This finding aligns with previous research indicating that blood transfusions remain a critical route for HBV transmission, especially in low‐resource settings where blood screening may be inconsistent [18]. Despite improvements in transfusion safety, the risk persists, necessitating enhanced blood safety protocols in oncology centers to mitigate this risk [19]. Implementing nucleic acid testing (NAT) could reduce residual risk, as demonstrated in settings where NAT adoption reduced HBV transmission by 80% [20].

The higher HBV prevalence in hematological malignancies (32%) compared to other cancers (e.g., breast malignancies at 14.3%) may reflect immunosuppressive therapies like rituximab, which increase reactivation risk. This aligns with global studies where lymphoma patients exhibit elevated HBV rates [9]. In contrast, lower rates in Egypt (8.5%) and Nigeria (14.2%) highlight regional disparities in screening and endemicity [21, 22].

The observed 10.3% HBV prevalence in Khartoum State is higher than rates in Asia (6%–8%) but aligns with Sub‐Saharan African averages (9%–12%) [13], reflecting persistent gaps in vaccination and blood safety protocols.

Additionally, household exposure to HBV was noted as a risk factor, with 16.1% of HBV‐positive participants reporting an infected household member. This supports existing literature on the significance of horizontal transmission within families, particularly in high‐prevalence areas [23]. Given that cancer patients may already be immunocompromised, the presence of HBV in the household poses an additional challenge, particularly during chemotherapy [24].

The potential for HBV reactivation during chemotherapy is a critical concern. Reactivation can lead to severe liver damage, complicating cancer treatment and worsening patient outcomes [25]. The high seroprevalence observed in this study reinforces the necessity for routine HBV screening and preemptive antiviral therapy for cancer patients prior to chemotherapy, as recommended by international guidelines [26].

The findings of this study highlight the urgent need for targeted HBV screening and vaccination efforts in cancer patients, particularly in regions with high endemicity [27]. Routine screening should be integrated into oncology care protocols to prevent HBV reactivation and manage infections proactively. For patients who test negative for HBsAg, vaccination should be considered, especially for those with identified risk factors such as blood transfusions or household exposure [28].

In conclusion, the seroprevalence of HBV among newly diagnosed cancer patients in Khartoum State reveals significant associations with demographic factors such as gender, age, and geographical location, as well as specific risk factors like blood transfusions and household exposure. These findings underscore the need for comprehensive HBV screening and preventive measures in this vulnerable population. Further research is warranted to explore the long‐term implications of HBV in cancer patients and to inform clinical practice guidelines aimed at reducing HBV‐related morbidity and mortality [29].

This study has several limitations that should be acknowledged. First, the cross‐sectional design captures data at a single point in time, making it difficult to assess causality between HBV infection and its potential reactivation during chemotherapy. Additionally, while the study sample of 300 patients is substantial, it may not fully represent all cancer patients in Sudan or account for regional variations in HBV prevalence. Furthermore, the reliance on self‐reported risk factors, such as a history of blood transfusion, may introduce recall bias, potentially affecting the accuracy of the data. Finally, this study did not test for anti‐HBc antibodies, which could provide critical insights into past HBV exposure and occult infections. Future studies should include anti‐HBc testing to better assess reactivation risks in chemotherapy patients.

## 5 Recommendations

Future studies should consider a longitudinal approach to better understand the progression and reactivation of HBV among cancer patients undergoing chemotherapy. Expanding the sample size and including a broader range of oncology centers across Sudan would improve the generalizability of the findings. Additionally, implementing routine HBV screening and adopting NAT for HBV DNA in Sudanese blood banks to minimize transfusion‐related infections, particularly in high‐burden regions, is recommended. It is also recommended that healthcare professionals receive ongoing training on HBV management in immunocompromised patients, ensuring early detection and intervention.

## 6 Conclusion

In conclusion, this study highlights a significant public health concern regarding the seroprevalence of HBV among newly diagnosed cancer patients in Khartoum State. The findings underscore the need for routine HBV screening prior to chemotherapy to prevent the serious complications associated with reactivation. Addressing key risk factors, such as a history of blood transfusion and household exposure, can enhance preventive measures. By implementing targeted interventions, healthcare providers can improve the safety and outcomes of cancer treatment in high‐risk populations, ultimately contributing to better patient care in Sudan.

## Ethics Statement

Ethical approval for the study was secured from the Sudan Medical Specialization Board, the Ministry of Health's Research Department in Khartoum State, and the participating oncology centers, including the Radiation and Isotopes Center Khartoum, Amal Tower, Tayba Oncology Center, and Shafi Oncology Center.

## Consent

Participants were informed about the study's purpose and their right to voluntary participation. All data were kept anonymous and confidential. Written informed consent was obtained from participants, with provisions for illiterate individuals to provide consent via fingerprint. Participants were also informed of their right to withdraw from the study at any time.

## Conflicts of Interest

The authors declare no conflicts of interest.

## Data Availability

The datasets generated and/or analyzed during the current study are available from the corresponding author upon reasonable request. Due to patient confidentiality and ethical restrictions, data access is subject to approval by the relevant ethics committee. Requests for data should be directed to abubakerabdalgafar@gmail.com.
